# Adult-Onset Periodic Fever, Aphthous Stomatitis, Pharyngitis, and Cervical Adenitis Syndrome on the Basis of Selective IgA Deficiency

**DOI:** 10.1155/2024/9845501

**Published:** 2024-07-31

**Authors:** Seda Altiner, Alper Ekinci

**Affiliations:** Department of Immunology and Allergy Ankara University Faculty of Medicine, Ankara, Türkiye

## Abstract

Periodic fever, aphthous stomatitis, pharyngitis, and cervical adenitis syndrome (PFAPA) is an autoinflammatory disease that is thought to occur with the contribution of genetic and environmental factors, but its etiology has not been clearly elucidated. It is characterized by recurrent attacks with fever, pharyngitis, oral aphthous lesions, and cervical lymphadenopathy, and an increase in the level of serum acute phase reactants is observed during the attacks. Although PFAPA usually begins in childhood, adult-onset cases are also reported in the literature. In the pathogenesis of PFAPA, an increase in the expression of various inflammatory cytokines, especially interleukin-1*β* (IL-1*β*), is observed as a result of the increase in inflammasome activity. Selective IgA deficiency (SIgAD) is the most prevalent primary immunodeficiency. Although most SIgAD cases remain asymptomatic and remain undiagnosed, it is known that the risk of mucosal infection is generally increased in SIgAD cases. In addition, the frequency of autoinflammatory diseases is increased in SIgAD cases compared with the general population. We aim to present a case of adult-onset PFAPA and SIgAD coexistence.

## 1. Introduction

Periodic fever, aphthous stomatitis, pharyngitis, and cervical adenitis syndrome (PFAPA) is an autoinflammatory disease characterized by attacks that usually begin in early childhood and is characterized by recurrent fever, pharyngitis, oral aphthous lesions, and cervical lymphadenopathy [1]. These attacks last 3–7 days and recur every 2–8 weeks [1]. PFAPA is the most common periodic fever syndrome in children not of Mediterranean origin [1]. Although the etiology of PFAPA has not been clearly elucidated, the accused factors include oropharyngeal microbial flora, polygenic predisposition that causes immune dysregulation, and infections [2]. During attacks, serum levels of interleukin-1*β* (IL-1*β*), IL-6, and IL-18 increase due to the increase in inflammasome activity. In addition, an increase in acute phase reactants such as erythrocyte sedimentation rate (ESR), C-reactive protein (CRP), and serum amyloid A (SAA) can be observed [2]. As a result of the increase in IL-1*β*, the development of a T helper 1 (Th1)-mediated inflammatory response is observed [2]. In this context, both innate immunity and adaptive immunity play a role in disease development [2].

Although PFAPA is primarily considered a pediatric disease, adult-onset cases are increasingly described in the literature [3]. The criteria defined by Cantarini et al. can be used in the diagnosis of adult-onset cases [4]. According to these criteria, recurrent fever, pharyngitis, and cervical adenitis must be present during the attack; however, the interattack period should be asymptomatic [4]. In addition, in adult-onset PFAPA cases, unlike pediatric cases, the presence of oral aphthous lesions is valuable for diagnosis, but it is not considered absolutely necessary [4]. Moreover, the various diseases that may cause similar findings, such as upper respiratory tract infections and other autoinflammatory disorders including familial Mediterranean fever (FMF), hyperimmunoglobulin D (IgD) syndrome (HIDS), tumor necrosis factor (TNF) receptor-associated periodic syndrome (TRAPS), cryopyrin-associated periodic syndrome (CAPS), and autoimmune diseases including Behçet's disease, Still's disease, Schnitzler's disease, and neoplastic diseases, should be excluded for diagnosis of PFAPA [4].

PFAPA treatment aims to alleviate acute attacks as well as reduce the frequency of attacks [5]. Nonsteroidal anti-inflammatory drugs (NSAIDs), a single dose of 1-2 mg/kg systemic corticosteroids, and IL-1 inhibitors can be used in the treatment of attacks [5]. Options such as cimetidine, colchicine, IL-1 inhibitors, vitamin D, and pidotimod can be used to prevent attacks [5]. Tonsillectomy can provide a cure, especially in pediatric cases, but since pediatric cases generally have a self-limited course, tonsillectomy is not considered as the primary treatment option [2]. Adult-onset cases do not benefit as significantly from tonsillectomy as pediatric cases [5].

Selective IgA Deficiency (SIgAD) is the most common primary immunodeficiency and is diagnosed by measuring the serum IgA level below 0.07 g/dL in an individual over 4 years of age without any other pathological immunodeficiency findings [6]. Although it is generally asymptomatic, an increase in the frequency of mucosal infections has been reported in SIgAD cases because IgA is responsible for mucosal immunity [6]. In addition, it is stated in the literature that the frequency of autoimmune diseases in SIgAD cases is significantly increased compared with the general population [6].

A few cases of the coexistence of PFAPA and immunodeficiency conditions have been previously described in the literature [7, 8]. We aim to present a case of adult-onset PFAPA accompanied by SIgAD.

## 2. Case Report

A 25-year-old female patient applied to our clinic with the complaint of high fever attacks that were accompanied by complaints of headache, fatigue, sore throat, difficulty in swallowing, and mouth sores, which recurred every 4-5 weeks and lasted for about 5 days. During the previous attacks, antibacterial treatments commenced with the diagnosis of bacterial pharyngitis, providing no relief. Bacteriological and virological examinations performed during her previous attacks were negative. With the preliminary diagnosis of recurrent upper respiratory tract infection (URTI), the patient was referred to us after low IgA levels were detected in the tests performed for the possibility of immunodeficiency in another center.

Apart from her current symptoms and findings, the patient did not describe any additional diseases or regular medication use. There was no history of any allergic disease, autoinflammatory disease, autoimmune disease, primary immunodeficiency condition, or neoplastic disease in the family members.

**Figure 1:**
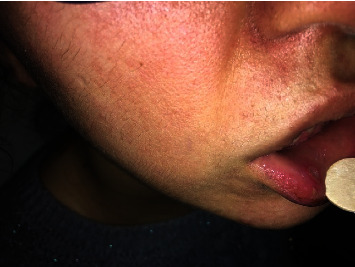
Oral aphthous lesions of the patient.

During the physical examination of the patient, the temperature was measured at 39°C. There were no abnormalities in the other vital signs. On examination of the oropharyngeal mucosa, the tonsils were hyperemic, and there were two aphthous lesions, one on the left tonsil and one on the inner side of the lower lip (Figure 1). There was a 4 cm-sized, painful cervical lymphadenopathy on the right side of the neck. The pathergy test was negative. Apart from these findings, there were no significant pathological findings on physical examination.

In laboratory examinations, the complete blood count, liver and kidney function tests, serum albumin and total protein levels, serum iron parameters, vitamin B12, and folic acid levels were normal. She had a high ESR (36 mm/h) and a very high CRP (69.2 mg/L).

Antistreptolysin O (ASO) and rheumatoid factor (RF) were found to be negative. Hepatitis B virus (HBV) surface antigen (HBsAg), HBV surface antibody (anti-HBs), antihepatitis C virus (anti-HCV), and antihuman immunodeficiency virus (anti-HIV) serological examinations were negative. Antinuclear antibody (ANA), antidouble-stranded deoxyribonucleic acid (anti-dsDNA), and antineutrophil cytoplasmic antibody (ANCA) were detected as negative. Cultures and polymerase chain reaction (PCR) tests for the detection of infectious agents, including URTI, urinary tract infection, Epstein–Barr virus (EBV), and cytomegalovirus (CMV), were negative (Table 1).

Immune work-up confirmed selective IgA deficiency. Other immunoglobulins and IgG subgroups were within normal ranges, excluding IgG2 deficiency. Flow cytometry revealed mild B lymphopenia, which was resolved in follow-ups (Table 2).

We prescribed a 60 mg single dose of prednisolone treatment with the preliminary diagnosis of PFAPA. Following steroid treatment, the fever resolved in 24 hours. CRP levels returned to normal within 72 hours. Aphthous ulcers regressed as well. However, the patient's serum IgA level remained low (0.699 g/L) in the symptom-free period. Approximately 6 weeks later, the patient returned to our clinic with findings similar to those from her first admission. The second attack also responded well to single-dose prednisolone treatment. Regular colchicine treatment was planned to prevent recurring attacks. Also, regular follow-up was planned for the possibility of the development of common variable immunodeficiency (CVID) on the basis of SIgAD. However, the patient discontinued follow-up.

## 3. Discussion

A pediatric case with PFAPA and SIgAD has previously been described in the literature [8]. To the best of our knowledge, this is the first case of adult-onset PFAPA accompanied by SIgAD.

In our case, genetic tests were not performed to exclude other periodic fever syndromes and immunodeficiency conditions. However, genetic testing is not a necessary condition for the diagnosis of adult-onset PFAPA [4]. Besides, in our case, the history of recurrent fever along with oral aphthous ulcers, noninfectious pharyngitis, and cervical lymphadenitis and the dramatic improvement in symptoms and findings with single-dose systemic corticosteroid treatment were evaluated clinically as compatible with PFAPA. There was no documented infectious agent when she was admitted to us or in her previous evaluations. There was no diarrhea, abdominal pain, arthritis, pleuritis, skin rash, or conjunctivitis, which would be clinically more compatible with the FMF, HIDS, TRAPS, and CAPS [9]. There were no clinical and laboratory findings compatible with Behcet's disease, systemic lupus erythematosus (SLE), ANCA-associated vasculitis, inflammatory bowel diseases, nutritional deficiencies, infectious diseases, or cyclic neutropenia, which may cause oral aphthous ulcer [10]. Accordingly, our case was compatible with the adult-onset PFAPA diagnostic criteria defined by Cantarini et al. [4].

Genetic predisposition is among the mechanisms accused of the increased inflammation in SIgAD [6]. It is stated that there may be a relationship between SIgAD and some autoimmune diseases, for instance, SLE, type 1 diabetes mellitus, and celiac disease, as a result of the presence of some HLA haplotypes [6]. Although a common HLA haplotype between PFAPA and SIgAD has not been described in the literature, considering that the genetic defects associated with both diseases have not yet been fully elucidated, it is possible that a relationship can be established between some HLA haplotypes and the coexistence of these two diseases in the future.

Interferon induced with helicase C domain 1 (IFIH1) is a gene that plays a role in creating a response to viral infections through the activation of nuclear factor kappa B (NF-*κ*B) by detecting double-stranded ribonucleic acid (dsRNA), whose expression increases as a result of type 1 interferon stimulation [11]. IFIH1 is among the non-HLA gene defects that display a significant association with SIgAD [11]. In addition, a relationship has been identified between IFIH1 and various autoimmune diseases, including SLE and type 1 diabetes mellitus [11]. In a recent study conducted on pediatric rheumatological diseases, IFIH1 gene variation was detected in one case diagnosed with PFAPA [12]. Accordingly, although genetic analysis was not performed in our case, one of the possible reasons for the coexistence of SIgAD and PFAPA may be an IFIH1 gene defect.

In a recent study, a decrease in Treg cells, which play a key role in the development of immune tolerance and prevent the development of excessive inflammation, was detected in SIgAD cases, and a correlation was found between the decrease in Treg cells and SIgAD disease severity, especially in SIgAD cases with autoimmunity [13, 14]. It is stated that Treg cells contribute to IgA production through transforming growth factor-*β* (TGF-*β*) secretion [14]. It is also stated that Treg cells may also be reduced in PFAPA cases [15]. It is denoted that the deficiency of various cytokines, including TGF-*β* as well as IL-4, may contribute to the pathogenesis of the disease in SIgAD cases [16]. In a recent study conducted on pediatric cases with PFAPA syndrome, it was found that the IL-4 serum level in PFAPA cases was significantly reduced compared with recurrent tonsillitis cases [17]. Furthermore, in a recent study conducted on a murine model, it was determined that IL-4 expression at a physiological level supports the immunomodulation carried out by Treg cells [18]. Thus, it can be concluded that further research is needed to question the relationships between Treg and IL-4 in the coexistence of PFAPA and SIgAD.

Another possibility is that the sensitivity of T and B cells to antigens increases as a result of mucosal infections, which increase in frequency in SIgAD and the disruption of mucosal barrier integrity [6]. There is no clearly identifiable infectious agent in the etiology of PFAPA; however, it is depicted that changes in the number and ratio of microflora elements of the oropharyngeal mucosa may trigger the disease, or disease findings may develop due to an excessive immune response to some triggering elements as a result of immune dysregulation [2]. In our case, no infectious agent could be detected before our patient's clinical findings appeared or during the time the clinical findings were present. Nevertheless, it is possible that the IgA deficiency in our case was present long before PFAPA-related findings appeared. It is possible that the mucosal barrier integrity may be disrupted and the susceptibility to the development of PFAPA may be increased as a result of various recurring infections that are dismissed by the patient and are self-limiting without requiring treatment.

Another possibility is that there are data indicating that IgA may have a suppressive effect on inflammatory pathways [19]. Fc alpha receptor (Fc*α*RI) is an IgA receptor found on myeloid lineage cells [19]. It is stated that an anti-inflammatory effect can occur through the immunoreceptor tyrosine-based activation motif (ITAM) as a result of monomeric IgA binding with Fc*α*RI [19]. Hence, the IgA deficiency itself may have contributed to the coexistence of SIgAD and PFAPA in our patient.

Despite all these possible mechanisms, it is possible that the simultaneous presence of PFAPA and SIgAD in our case is coincidental. Further research is needed to fully understand this matter.

## 4. Conclusion

Inborn errors of immunity, including SIgAD, predispose patients to hyperinflammation. This report aims to raise awareness of hyperinflammation accompanying a diagnosis of primary immunodeficiency. To the best of our knowledge, our case represents the first documented patient in the literature of the coexistence of SIgAD and adult-onset PFAPA.

## Figures and Tables

**Table 1:** Initial laboratory evaluation of the patient.

Parameter	Patient's value	Reference values
White blood cell (×10^9^/L)	7.42	4.5–11
Neutrophil (×10^9^/L)	4.62	1.8−7.7
Lymphocyte (×10^9^/L)	1.93	1.5−4
Hemoglobin (g/dL)	13.6	11.7–15.5
Platelet (×10^9^/L)	275	150–400
ESR (mm/h)	**36**	0–20
CRP (mg/L)	**69.2**	0–5
Alanine aminotransferase (U/L)	9	0–35
Aspartate aminotransferase (U/L)	12	0–35
Blood urea nitrogen (mg/dL)	7	6–20
Creatinine (mg/dL)	0.67	0.5−0.9
Albumin (g/L)	46	35–52
Total protein (g/L)	81	64–83
Ferritin (ng/mL)	37.2	11–306.8
Vitamin B12 (pg/mL)	187	126.5–505
Folic acid (ng/mL)	7.87	5.9–24.8
C3 (g/L)	1.42	0.79−1.52
C4 (g/L)	0.315	0.16−0.38
ASO (IU/mL)	23	0–200
RF (IU/mL)	<10	0–14
ANA	Negative	Negative
Anti-dsDNA	Negative	Negative
ANCA	Negative	Negative
HBsAg	Negative	Negative
Anti-HBs	Negative	Negative
Anti-HCV	Negative	Negative
Anti-HIV	Negative	Negative
Throat swab culture	Negative	Negative
Urine culture	Negative	Negative
EBV PCR	Negative	Negative
CMV PCR	Negative	Negative
URTI PCR	Negative	Negative

*p* < 0.05 for both the bold values. These values show inflammatory situation of the patient.

**Table 2:** Immune work-up of the patient.

Parameter	Patient's value	Reference values
IgG (g/L)	13.8	7.51−15.6
IgG1 (g/L)	6.05	3,824−9,286
IgG2 (g/L)	**7.30**	2,418−7,003
IgG3 (g/L)	1.10	0.218−1,761
IgG4 (g/L)	0.352	0.039−0.864
IgA (g/L)	**0.687**	0.82−4.53
IgM (g/L)	1.73	0.46−3.04
IgE (kU/L)	18.9	—
Lymphocyte (×10^9^/L)	1.93	1.5−4
CD3+ T cells (%)	78	60–85
CD4+ T helper (%)	57	29–59
CD8+ T cytotoxic (%)	37	19–48
B lymphocyte (%)	**6**	7–23
Natural killer cells (%)	16	6–29

The bold values indicate that our patient had selective IgA deficiency; thus, the value of IgA was lower than the lower limit of the reference values. The percentage of B lymphocyte and the value of IgG2 were not clinically significant.

## Data Availability

The data used to support the findings of this study are available from the corresponding author upon reasonable request.
